# Facilitated Tau Degradation by USP14 Aptamers via Enhanced Proteasome Activity

**DOI:** 10.1038/srep10757

**Published:** 2015-06-04

**Authors:** Jung Hoon Lee, Seung Kyun Shin, Yanxialei Jiang, Won Hoon Choi, Chaesun Hong, Dong-Eun Kim, Min Jae Lee

**Affiliations:** 1 Department of Biochemistry and Molecular Biology, Neuroscience Research Institute, Seoul National University College of Medicine, Seoul 110-799, Republic of Korea; 2 Department of Applied Chemistry, College of Applied Sciences, Kyung Hee University, Yongin 446-701, Republic of Korea; 3 Department of Bioscience and Biotechnology, Konkuk University, Seoul 143-701, Republic of Korea

## Abstract

The ubiquitin-proteasome system (UPS) is the primary mechanism by which intracellular proteins, transcription factors, and many proteotoxic proteins with aggregation-prone structures are degraded. The UPS is reportedly downregulated in various neurodegenerative disorders, with increased proteasome activity shown to be beneficial in many related disease models. Proteasomes function under tonic inhibitory conditions, possibly *via* the ubiquitin chain-trimming function of USP14, a proteasome-associated deubiquitinating enzyme (DUB). We identified three specific RNA aptamers of USP14 (USP14-1, USP14-2, and USP14-3) that inhibited its deubiquitinating activity. The nucleotide sequences of these non-cytotoxic USP14 aptamers contained conserved GGAGG motifs, with G-rich regions upstream, and similar secondary structures. They efficiently elevated proteasomal activity, as determined by the increased degradation of small fluorogenic peptide substrates and physiological polyubiquitinated Sic1 proteins. Additionally, proteasomal degradation of tau proteins was facilitated in the presence of the UPS14 aptamers *in vitro*. Our results indicate that these novel inhibitory UPS14 aptamers can be used to enhance proteasome activity, and to facilitate the degradation of proteotoxic proteins, thereby protecting cells from various neurodegenerative stressors.

Proteasomes are the primary proteolytic machinery used by cells for homeostasis of various regulatory proteins. This catabolic process is mainly mediated by ubiquitin (Ub)-dependent proteolysis, but when the proteins are intrinsically disordered proteins, they can be degraded by proteasomes in a Ub-independent manner^1^. The 26S proteasome is a multimeric complex, composed of a 28-subunit core particle (CP or 20S complex) and a 19-subunit regulatory particle (RP, PA700, or 19S complex)^2^. The RP recognizes the polyubiquitin (polyUb) chains of its substrates, deubiquitinates them before proteasomal proteolysis is initiated, and translocates the substrates to the catalytic cavity of the CP.

The ubiquitin-proteasome system (UPS) appears to have its own quality control mechanisms, one of which is accomplished by the proteasome-associated deubiquitinating enzymes (DUBs) USP14 and UCH37^3^. UPS14 interacts with RPN1, while UCH37 binds to ADRM1/RPN13^4^,^5^. Both are located on the RP, relatively far from the substrate entry pore and the Ub receptors RPN1 and RPN10. UPS14 and UCH37 are thought to mediate stepwise disassembly of the Ub chain from the distal end^6^. This “chain-trimming” effect can delay proteasomal degradation by weakening the interaction between the Ub receptors of the proteasomes, and the polyUb chains of the substrates. Deletion of the *USP14* gene, or chemical treatment with USP14 inhibitors, results in accelerated proteasomal degradation of various target substrates^7^. These findings suggest that USP14 is a potential therapeutic target for treating diseases where toxic proteins accumulate. However, it has also been reported that the trimming of Ub chains might promote proteasomal degradation^8^. The mechanism regulating this remains to be elucidated, but probably involves the rate of Ub chain-trimming on the proteasome in coordination with substrate translocation.

Aptamers are molecules composed of single-stranded nucleic acids (15–50 bases) that have been generated by an *in vitro* selection process from a large pool of random sequences. This technique is known as systemic evolution of ligands by exponential enrichment (SELEX)^9^,^10^. Since the introduction of SELEX technology, a wide range of biological targets, including small molecules, peptides, proteins, nucleic acids, cells, tissues and organisms, have been reported to bind to aptamers with high specificity. Aptamers are often referred to as “chemical antibodies,” and are one of only a few classes of biomolecules that can be manufactured to bind to multiple different targets. RNA aptamers have been isolated and shown to have stable conformations *in vivo*, following some modification. They can specifically bind to proteins such as human immunodeficiency virus Tat^11^, reverse transcriptase^12^, hepatitis C virus NS3 protease/helicase^13^,^14^, NS5B RNA-dependent RNA polymerase^15^, and severe acute respiratory syndrome NTPase/helicase^16^. In addition, RNA aptamers can bind to prostate cancer cells through the extracellular portion of the prostate-specific membrane antigen^17^, and to brain tissue^18^.

Aptamers that bind to specific proteins can repress the enzymatic activity of those proteins or protein-protein interactions. The active sites or interacting motifs usually offer more exposed heteroatoms, which mediates hydrogen bonds or other strong interactions with the aptamers^19^. For therapeutic applications, inhibitory aptamers are often chemically modified to be resistant to degradation mediated by serum. The age-related macular degeneration drug pegaptanib is a 27-nt aptamer that targets vascular endothelial growth factor. It is conjugated with 40 kDa polyethylene glycol and contains inverted nucleotides at the 3′ terminus^20^. Considering the rapid progress in aptamer biology and related technologies, aptamers are now considered essential for understanding and modulating various pathophysiological processes.

To overcome the limitation of small-molecule USP14 inhibitors, we identified three novel USP14-binding RNA aptamers that suppressed the deubiquitinating activity of USP14 *in vitro*. Consistent with the effects of USP14 upon the proteasome, these USP14 aptamers enhanced proteasome activity, and facilitated the degradation of Alzheimer’s disease (AD)-implicated tau proteins. The inhibitory USP14 aptamers were non-cytotoxic and effectively relieved proteopathic stress in cultured cells. Therefore, UPS14 aptamers could offer an interesting alternative to delay the aggregation process of toxic, aggregation-prone proteins.

## Methods

### Purification of recombinant USP14 proteins

**Figure 1:**
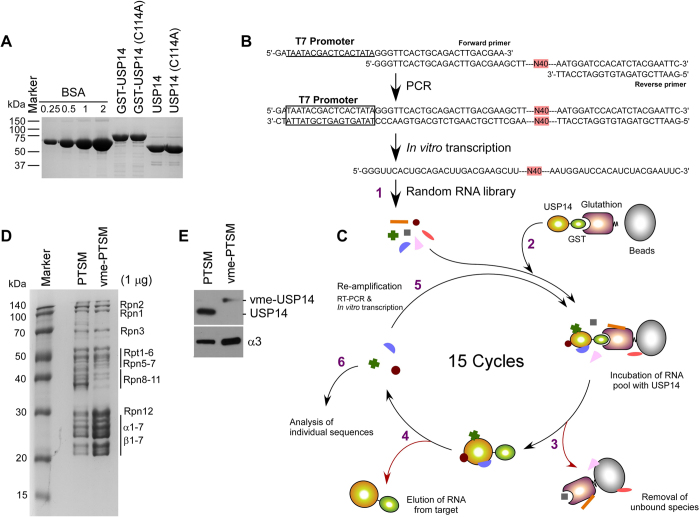
Purification of USP14 and human proteasomes, preparation of vme-proteasomes, and SELEX for USP14 aptamers. (**A**) Approximately 1 μg of purified recombinant USP14, USP14(C114A), GST-USP14, and GST-USP14(C114A) was analyzed by sodium dodecyl sulfate polyacrylamide gel electrophoresis (SDS-PAGE) and Coomassie Brilliant Blue (CBB) staining. Bovine serum albumin (BSA) was used as a standard. (**B**) RNA sequences used for *in vitro* selection. The random RNA library contained random 40-nt sequences, flanked by a 3’ region (23 bp) and a sequence containing the T7 promoter (46 bp, underlined). (**C**) Scheme for the SELEX strategy. (1) Purified RNAs were incubated with GST-USP14. (2) USP14-RNA complexes were captured by glutathione agarose beads. (3) Unbound RNA molecules were removed by centrifugation. (4) USP14-RNA complexes were dissociated with elution buffer containing excess imidazole. (5) RNAs bound to USP14 were prepared by phenol:chloroform extraction and ethanol precipitation. Recovered RNAs were reverse transcribed, amplified by polymerase chain reaction (PCR), and *in vitro* transcribed. (6) Following 15 rounds of selection, the resultant cDNA was amplified and cloned into pcDNA 3.1. (**D**) Human proteasomes (PTSM) were purified from β4-tagged HEK293 cell lines. Some of the proteasomes were treated with 1 μM ubiquitin-vinylmethylester (Ub-vme) to yield vme-PTSMs. PTSMs (1 μg) and vme-PTSMs (1 μg) were separated on 12% gradient gels and stained with CBB. (**E**) Western blotting analysis of USP14 and proteasome subunit α3. Approximately 250 ng of PTSMs or vme-PTSMs were subjected to SDS-PAGE. Cropped gels/blots are used in (E).

We transformed pGEX-2T, pGEX-2T-USP14, and pGEX-2T-USP14(C114A) into *Escherichia coli* strain BL21 (DE3). We purified USP14 and USP14(C114A) and their glutathione S-transferase (GST)-conjugated counterparts [GST-USP14 and GST-USP14(C114A), respectively] using previously described methods^7^, with some modifications. Cultures were incubated at 37 °C, and when the optical density at 600 nm (OD_600_) was 0.6–0.8, we added IPTG to each culture (final concentration of 1 mM), and allowed cultures to incubate overnight at 25 °C. Cells were harvested in phosphate-buffered saline (PBS) containing protease inhibitor cocktails and lysed by sonication. Following centrifugation, lysates were filtered and supernatants incubated with GST sepharose 4B resin (GE Healthcare, Little Chalfont, UK) at 4 °C for 1 h. After washing with PBS, GST-USP14 was eluted using 10 mM reduced glutathione (50 mM Tris-HCl pH 8.0). The GST tag was removed by incubating protein fractions with thrombin in a cleavage buffer [50 mM Tris-HCl pH 8.0, 150 mM NaCl, 2.5 mM CaCl_2_, and 0.1% (v/v) 2-mercaptoethanol] for 3 h at room temperature (RT). After centrifugation, supernatants were incubated with benzamidine sepharose resin (GE Healthcare) to remove any residual thrombin. Purified GST (data not shown), GST-USP14, GST-USP14(C114A), USP14, and USP14(C114A) were separated by sodium dodecyl sulfate polyacrylamide gel electrophoresis (SDS-PAGE), and gels were stained with Coomassie Brilliant Blue R-250 (CBB) to determine the size and purity of proteins (Fig. 1A).

### *In vitro* selection of RNA aptamers

A random RNA library was generated as previously described^21^ using polymerase chain reaction (PCR), T7 *in vitro* transcription, and a DNA template (109 bp) containing 40 random nucleotides. Briefly, the aptamer library template DNA (5′-GGG TTC ACT GCA GAC TTG ACG AAG CTT -40 N-A ATG GAT CCA CAT CAT CTA CGA ATT C-3′) was generated by PCR using a forward primer (5′-GAT AAT ACG ACT CAC TAT AGG GTT CAC TGC AGA CTT GAC GAA-3′) containing the T7 promoter sequence (underlined) and a reverse primer (5′-TTA CCT AGG TGT AGA TGC TTA AG-3′) (Fig. 1B). The RNA aptamer library was prepared using T7 RNA polymerase in *in vitro* transcription buffer (50 mM Tris-HCl pH 7.5, 15 mM MgCl_2_, 2 mM spermidine, 5 mM DTT, and 2 mM NTPs) at 37 °C for 4 h. Products were treated with DNase I (Thermo Scientific) at 37 °C for 30 min, subjected to 12% denaturing urea PAGE using, and purified from gels. A 6-μg aliquot of each generated RNA pool was incubated with 100 μL of GST sepharose 4B resin in binding buffer [30 mM Tris-HCl pH 7.5, 150 mM NaCl, 1.5 mM MgCl_2_, 2 mM DTT, and 1% (w/v) bovine serum albumin (BSA)] for 30 min at RT with occasional shaking. The RNA-bead complexes were transferred to porous centrifuge columns (Pierce), and RNA pools that were unbound to sepharose resin were collected by centrifugation. The same procedure was performed using 2 μg of GST proteins to remove RNAs that were nonspecifically bound to GST or glutathione resin. During each round of selection, GST-tagged proteins were incubated with 6 μg of pre-cleared RNA pools in 100 μL of binding buffer for 30 min at RT, and then with 100 μL of GST sepharose 4B resin for an additional 30 min at RT. The quantity of GST-tagged proteins was gradually decreased (2 μg, rounds 2–8; 1 μg, round 9; 0.5 μg, rounds 10–11; 0.25 μg, rounds 12–13; and 0.125 μg, rounds 14–15) to achieve more stringent conditions. The RNA-GST-USP14 complexes were then washed three times with PBS, and eluted with 20 mM glutathione in binding buffer. Eluted RNAs were purified by phenol:chloroform extraction and ethanol precipitation, reverse-transcribed with AccuPower RT PreMix (Bioneer), and amplified by PCR. The amplified cDNA was purified using the AccuPrep Gel purification kit (Bioneer) and transcribed with T7 RNA polymerase for the next round of selection (Fig. 1C).

### Cloning and sequencing

After 15 rounds of selection, the resulting cDNA was PCR-amplified using forward (5′-GAT AAT ACG ACT CAC TAT AGG GTT CAC TGC AGA CTT GAC GAA-3′) and reverse (5′-TTA CCT AGG TGT AGA TGC TTA AG-3′) primers. Amplicons were digested with *Hind*III and *Eco*RI, and cloned into the pcDNA 3.1 vector (Invitrogen) for sequencing. The secondary structure of selected RNA aptamers was predicted by the Mfold program based on the Zuker algorithm (http:\\mfold.rna.albany.edu). For subsequent activity testing and inhibition assays, individual RNA aptamers were amplified by PCR using forward (5′-ATT AAT ACG ACT CAC TAT AGG G-3′) and reverse (5′-TTA CCT AGG TGT AGA TGC TTA AG-3′) primers, and *in vitro* transcribed using T7 RNA polymerase. We used 40-nt sequences that were irrelevant to USP14 aptamers as controls (UUU GUC UAG CGC GUA GUG GGG AGA UGU UGU GAU ACU GGG G).

### Measurement of RNA aptamer binding to USP14

We loaded and immobilized 2 pmol of GST-USP14 or GST onto a GST sepharose 4B resin, and then added 20 pmol of RNA aptamers. The RNA eluted from the resin was reverse-transcribed and the resulting cDNA subjected to PCR. Agarose gel electrophoresis, followed by ethidium bromide staining, was carried out to visualize protein-bound RNA in 2% (w/v) agarose gels. For cloning, we used primers USP14-1 mutant (5′-GCA GTG ATG TGC TTC TAA AAA ACA ACC TAA AAA AAT TGC C-3′), USP14-2 mutant, (5′-CTG AAA AAA AGT TAG TTT CGC TGG TTT AAA ATC GGT GCG G-3′), and USP14-3 mutant (5′-AAA AAA AAG GCT CGT TTG GCC TGC CGA AAA AAG GCC GGG A-3′). The USP14 aptamer mutants were generated by insertion of corresponding oligonucleotides into the pcDNA 3.1 vector, amplified by PCR using the same forward and reverse primers, and *in vitro* transcribed using T7 RNA polymerase as described above.

### Purification of human proteasomes and preparation of vinylmethylester-proteasomes

Human proteasomes were purified by affinity chromatography from a stable HEK293 cell line harboring biotin-tagged human β4 as previously described^7^, with some modifications. Cells were cultured in 15-cm dishes, harvested in lysis buffer [50 mM NaH_2_PO_4_ pH 7.5, 100 mM NaCl, 10% glycerol, 5 mM MgCl_2_, 0.5% NP-40, 5 mM ATP, and 1 mM DTT] containing protease inhibitors, and homogenized using a Dounce homogenizer. After centrifugation, supernatants were incubated with streptavidin agarose resin (Millipore) for 5 h at 4 °C. Beads were washed with lysis buffer and TEV buffer (50 mM Tris-HCl pH 7.5, 1 mM ATP, and 10% glycerol). The 26S proteasome was eluted from the resin by incubating with TEV protease (Invitrogen) in TEV buffer for 1 h at 30 °C, and concentrated using an Amicon ultra-spin column (Millipore). To inhibit the deubiquitinating activity of proteasomes, ubiquitin-vinylmethylester (Ub-vme; LifeSensors) was added as previously described^22^. Residual Ub-vme was removed by washing the beads three times with 50 bed volumes of washing buffer. Proteasomes (240 ng) were separated by SDS-PAGE using a 4–20% gradient gel. Gels were stained using the EzWay silver staining kit (Koma Biotech) or CBB. The obtained proteasomes, which we designated vme-proteasomes, were tested to confirm the elimination of deubiquitinating activity using the Ub-rhodamine 110 hydrolysis assay (Ub-rho110, LifeSensors) (data not shown).

### USP14 binding to proteasomes

The effect of USP14 aptamers on the interaction of USP14 with the proteasome was investigated using proteasome affinity pulldown assays. After HEK293 cells were harvested in lysis buffer, cell lysates were homogenized using a 1 mL syringe with a 26G × 1/2′′ needle. After centrifugation, supernatants were incubated with RNA aptamers for 10 min, and then with streptavidin agarose resin (Millipore) for 5 h at 4 °C. Beads were washed with lysis buffer and boiled in SDS sample buffer. The binding of USP14 to the 26S proteasome was monitored by western blotting using an antibody against USP14 (Bethyl Laboratories, Inc).

### Activity of proteasomes and DUBs

Based on the hydrolysis of fluorogenic succinyl-Leu-Leu-Val-Tyr-7-amido-4-methylcoumarin (suc-LLVY-AMC) peptides, the chymotrypsin-like activity of proteasomes was measured to determine their proteolytic activity. A suc-LLVY-AMC hydrolysis assay was carried out using purified proteasome and 12.5 μM suc-LLVY-AMC (Enzo Life Sciences) in assay buffer (50 mM Tris-HCl pH 7.5, 1 mM EDTA, 1 mg/mL BSA, 1 mM ATP, and 1 mM DTT). The Ub-rho110 hydrolysis reaction was carried out using proteasomes or USP14 activated by vme-proteasomes, along with 20 nM or 100 nM Ub-rho110 in the presence or absence of 1 μg/mL (equivalent to 33 nM) RNA aptamers. RNA aptamers were incubated with proteasomes for 5 min before they were added to substrates. To examine the effects of RNA aptamers on USP14, RNA aptamers were incubated with USP14 for 5 min, then with vme-proteasomes for 5 min, before they were added to substrates. UCHL3 and USP47 were kindly provided by Eunice Eun-Kyeong Kim; USP5 was provided by Kyeong Kyu Kim. Proteasomal activity and deubiquitinating activity were monitored by measuring free AMC or rho110 fluorescence, respectively, in black 96-well plates using a TECAN infinite m200 fluorometer.

### Kinetic analysis of USP14-mediated deubiquitinating activity

For kinetic analysis, the Ub-rho110 hydrolysis activity of USP14 on USP14 aptamers was monitored over a concentration course. Normalization of fluorescence intensity to the concentration of rhodamine 110 was achieved using a free rhodamine 110 standard curve (Santa Cruz Biotechnology). The *K*_*M*_ and *k*_cat_ parameters were determined by nonlinear regression fit, with a model derived from the Michaelis-Menten equation, using GraphPad Prism 5 (GraphPad Inc., USA)

### Ubiquitination of recombinant Sic1 and tau

Polyubiquitinated Sic1 with PY motifs (Ub-Sic1) was prepared as previously described^23^, with some modifications. The Ub conjugation reaction was conducted by incubating 10 pmol of Sic1^PY^, 2 pmol of Uba1, 5 pmol of Ubc4, 5 pmol of Rps5, and 1.2 nmol of Ub in a buffer (50 mM Tris-HCl pH 7.4, 100 mM NaCl, 1 mM DTT, 5 mM ATP, and 10 mM MgCl_2_) for 4 h at 25 °C. Conjugates were absorbed to a Ni-NTA resin (Qiagen, Germany), washed with buffer (50 mM Tris-HCl pH 8.0, 50 mM NaCl, and 40% glycerol), eluted with 200 mM imidazole in wash buffer, and dialyzed into wash buffer containing 10% glycerol. Recombinant tau and CHIP proteins were expressed and purified from pET29b vectors using conventional purification methods. Unphosphorylated tau (0.5 μg) was incubated with 200 ng of Uba1, 4 μg of UbcH5b, 2 μg of CHIP, 1 μg of HSP70, and 10 μg of Ub for 2 h at RT in a 100-μL reaction.

### *In vitro* degradation assays

Purified human proteasomes (5 nM) were incubated with 20 nM Ub-Sic1 or Ub-tau in proteasome assay buffer (50 mM Tris-HCl pH 7.5, 100 mM NaCl, 10% glycerol, 2 mM ATP, 10 mM MgCl_2_, and 1 mM DTT). In certain instances, USP14 was incubated with proteasomes for 5 min before reactions were initiated. To examine the effects of RNA aptamers on *in vitro* degradation assays, aptamers were incubated with USP14 for 5 min before USP14 was added to proteasomes. Reactions were terminated by adding 2 × SDS-PAGE sample buffer and subjected to SDS-PAGE. Ub-Sic1 and Ub-tau degradation was monitored by immunoblotting, using antibodies against T7 (Millipore) and tau (clone Tau-5, Invitrogen), respectively.

### Cell culture and RNA aptamer transfection

All cells in this study were cultured in Dulbecco’s modified Eagle’s medium supplemented with 10% fetal bovine serum. USP14 aptamer transfection was performed in 6-well plates using Lipofectamine 2000 (Invitrogen). Cells were transfected for 6 h and whole cell extracts were collected 24 h after the media was changed.

### Assessment of cell viability

Cell viability was assessed using a modified 3-(4,5-dimethylthiazol-2-yl)-2,5-diphenyltetrazolium bromide (MTT) assay^24^. HeLa cell cultures that were approximately 90% confluent in 96-well plates were treated with control or USP14 aptamers complexed with Lipofectamine 2000 at various concentrations (up to 50 μg/mL) for 4 h. We then added 10 μL of MTT solution to each well and incubated plates at 37 °C/5% CO_2_ for 2.5 h. Media was discarded and 200 μL of dimethyl sulfoxide was added to each well and plates were incubated for 30 min at RT. The absorbance of the solution at 570 and 630 nm was determined. Triplicate wells were assayed for each condition. Cell survival was also assessed when tau was induced by Dox, and when oxidative stress was induced by paraquat. Inducible tau cell lines were incubated with Dox (250 pg/mL) and paraquat (1 mM) for 3 h and then transfected with USP14 aptamers.

### Tau degradation assays

An inducible tau cell line (HEK293-trex-htau40)^25^ was transfected with USP14 aptamers and treated with various concentrations of Dox. After 24 h, whole-cell lysates were prepared in RIPA buffer and used for immunoblotting. Where necessary, cells were treated with 80 μg/mL cycloheximide (CHX) (Enzo Life Sciences) before harvesting. For SDS-PAGE, each lane was loaded with the extract from an equal cell number, generally corresponding to 10-20 μg/lane, or 1/10 of the sample recovered from one well of a 6-well plate.

## Results

### Preparation of recombinant USP14 proteins and selection of RNA aptamers

We previously showed that yeast and mammalian USP14, a proteasome-associated DUB enzyme, functions as an endogenous inhibitor of proteasomes^6^,^7^. The Ub chain-trimming effect of USP14 might antagonize the degradation of many proteasome substrates, with small molecule inhibitors facilitating proteolysis. However, small molecule inhibitors exhibit relatively high cytotoxicity and low inhibitory efficacy^7^. To identify USP14 aptamers, we used SELEX techniques with GST-USP14 (78 kDa), while USP14 (53 kDa) was also expressed and purified for subsequent analysis with aptamers and proteasomes (Fig. 1A).

To select for RNA aptamers with a high affinity that were specific to USP14, we generated a pool of RNAs (approximately 10^14^ molecules) containing a 40 nt random core sequence that was flanked by the T7 promoter site at the 5′ end and primer-binding sites at the 3′ end (Fig. 1B)^16^. Prior to initial screening, we performed negative selection over 15 rounds to remove non-specific RNAs bound to the glutathione-sepharose resin or to GST proteins from the RNA pool (Fig. 1C). The stringency of RNA binding to the USP14 protein was increased as the rounds progressed (*see* Methods).

Human 26S proteasomes were purified from a HEK293-derived cell line that stably expressed polyhistidine- and biotin-tagged β4 subunits^26^. Purified proteasomes showed an approximate 1:1 molar ratio between the RP and CP complexes (Fig. 1D). Basal deubiquitinating activity of USP14 is strongly activated by proteasomes, although the subunit responsible for activation remains to be elucidated^4^,^21^. The majority of proteasomal deubiquitinating activity was irreversibly blocked using Ub-vme^5^, which forms an adduct with Cys in the active site of the thiol protease DUB. The resulting vme-proteasomes appeared to retain the components of normal 26S proteasomes (Fig. 1D). Large amounts of USP14 were co-purified with the RP of proteasomes (Fig. 1E). When covalently modified by Ub-vme, the electrophoretic mobility of USP14 was reduced by 7.5 kDa, which corresponded to the size of Ub (Fig. 1E), which indicates that the DUB enzymes on the proteasomes, including USP14, were fully inactivated.

### Identification of USP14 aptamers and their inhibitory effects on deubiquitinating activity

**Figure 2:**
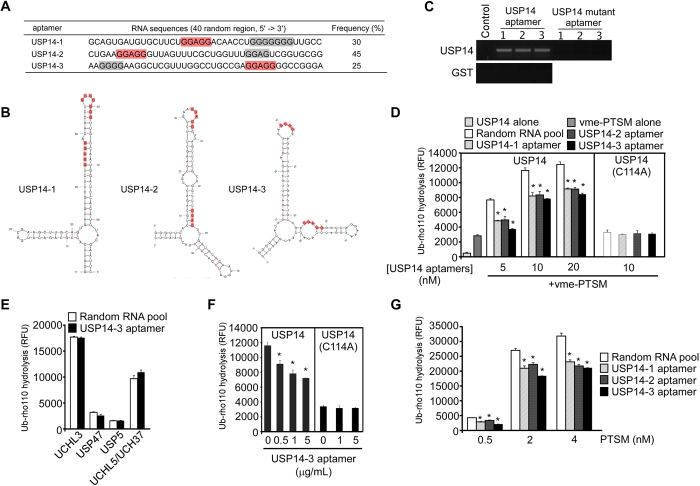
USP14-specific aptamers, predicted secondary structures, and inhibition of USP14 deubiquitinating activity. (**A**) Three different sequences of RNA aptamers were identified from the random RNA pool by SELEX. All sequences contained a conserved GGAGG motif (red) and a G-rich region (grey). (**B**) The secondary structures of the USP14 aptamers were calculated using Mfold. (**C**) Validation that the USP14 RNA aptamers bound to USP14. Control, 40 nt RNA oligonucleotides containing a sequence that was irrelevant to USP14 aptamers. (**D**) Ub-rho110 hydrolysis by recombinant USP14 or USP14(C114A). Prior to activation by vme-PTSM (1 nM), various concentrations of USP14 proteins were pre-incubated with RNA aptamers (1 μg/mL). As a control, 1 μg/mL random RNA pools were used. Note that USP14 alone (without activation by proteasomes) showed only basal Ub-rho hydrolysis activity. (**E**) Ub-rho110 hydrolysis by various deubiquitinating enzymes (DUBs) in the presence or absence of USP14 aptamers (1 μg/mL). (**F**) Ub-rho110 hydrolysis as in (D), except that USP14-3, at various concentrations, was incubated with USP14 (10 nM) or USP14(C114A) (10 nM) and vme-PTSM (1 nM). (**G**) The deubiquitinating activity of USP14 was monitored by Ub-rho110 (20 nM) hydrolysis in the presence of PTSMs and 1 μg/mL USP14 aptamers. The original RNA aptamer library (random RNA pool) was used as a control. All data represent the mean ± SD from three independent experiments. *p < 0.01 (one-way analysis of variance ANOVA with Bonferroni’s multiple comparison test). RFU, relative fluorescence unit. PTSM, 26S human proteasomes. vme-PTSM, Ub-vme-treated 26S human proteasomes.

We examined the RNA sequences of 20 randomly selected clones, and identified three different aptamers. These RNA aptamers were designated USP14-1, USP14-2, and USP14-3, and represented 30 (6/20), 45 (9/20), and 25% (5/20) respectively, of the identified aptamers (Fig. 2A). All of the aptamers contained conserved sequences and similar structural motifs, as predicted by Mfold^27^. In addition to two stem-loops in their secondary structures (Fig. 2B), there was a sequence of five conserved nucleotides (GGAGG), followed by a G-rich sequence. Our results suggest that the exposed GGAGG and G-rich sequences in RNA aptamers could be important for interactions with USP14.

None of the USP14 RNA aptamers was found to bind to GST-tagged proteins from the binding assay similar to the SELEX strategy (Fig. 2C), however they all exhibited a high affinity to USP14. The control RNA aptamers did not bind to USP14 (Fig. 2C). Mutations in the consensus GGAGG and G-rich sequences in the USP14 RNA aptamers abolished their ability to bind to USP14 (Fig. 2C). These findings suggest that the exposure of GGAGG and G-rich sequences in RNA aptamers is critical for their interaction with USP14.

To investigate the effects of USP14 aptamers on USP14-mediated deubiquitinating activity, the RNA aptamers were transcribed and purified *in vitro*. We used recombinant USP14 proteins and vme-proteasomes for hydrolysis of Ub-rho110, a highly sensitive fluorogenic substrate of DUBs. In the absence of vme-proteasomes, USP14 exhibited little deubiquitinating activity. In contrast, USP14 was activated in the presence of vme-proteasomes, exhibiting higher Ub-rho110 hydrolysis activity than for vme-proteasomes alone (Fig. 2D). The addition of USP14-1, USP14-2, or USP14-3 to the reaction resulted in a decrease in USP14 deubiquitinating activity (Fig. 2D and Supplementary Fig. 1). Inhibition of deubiquitinating activity by USP14 aptamers was specific to USP14, with the USP14-3 aptamer failing to inhibit the DUB activity of UCHL3, USP47, USP5, and UCHL5/UCH37 (Fig. 2E). USP14-3 inhibited USP14 deubiquitinating activity in a dose-dependent manner (Fig. 2F and Supplementary Fig. 2). The catalytically inactive mutant of USP14, USP14(C114A), exhibited little deubiquitinating activity and was unaffected by the USP14 aptamers (Fig. 2D,F), indicating that the USP14 aptamers specifically inhibit USP14 DUB activity.

Using Ub-rho110, we also measured the enzyme kinetics of recombinant USP14 with vme-proteasomes in the presence and absence of the aptamers (Supplementary Fig. 3). The *K*_M_ of USP14 bound to the aptamers (1716 ± 300 nM) was significantly higher than that for free USP14 (1124 ± 248 nM). However, *k*_*cat*_ values were comparable regardless of whether USP14 aptamers were present (0.948 ± 0.079 s^−1^ for USP14-3 *vs.* 0.938 ± 0.073 s^−1^ for control RNA aptamers), suggesting that the USP14 aptamers might affect substrate binding to USP14 to a greater extent than deubiquitinating activity. Further work will be required to determine the mechanism of inhibition of the USP14 aptamers. Our results strongly indicate that the newly identified USP14 aptamers effectively inhibit the deubiquitinating activity of USP14 *in vitro*.

We tested whether the total deubiquitinating activity of the proteasome was affected by the three USP14 aptamers. Proteasomes that were not treated with Ub-vme showed significant deubiquitinating activity, possibly from USP14 and UCH37, another proteasomal DUB that interacts with RPN13^3^ (Fig. 2G and Supplementary Fig. 4). The enzymatic redundancy between these two enzymes on the proteasome is unclear, but is considered insignificant because UCH37 exhibits much weaker Ub hydrolysis activity than USP14^7^. Addition of the USP14 aptamers strongly inhibited proteasome-mediated Ub-rho110 hydrolysis (Fig. 2G and Supplementary Fig. 4). USP14-3 showed the strongest inhibitory activity for all tested proteasome concentrations. The strong inhibition exhibited by USP14-3 could be a result of its ability to simultaneously bind to UCH37 (data not shown). This would indicate that UCH37 plays a more significant role in deubiquitinating polyubiquitinated substrates than previously thought^3^. Further study is required to determine whether chain-trimming by UCH37 can suppress proteasome activity as effectively as USP14. Taken together, our results provide strong evidence that USP14 RNA aptamers block the activity of USP14 and proteasomal DUBs.

### RNA aptamers accelerate proteasomal degradation of substrates *in vitro*

**Figure 3:**
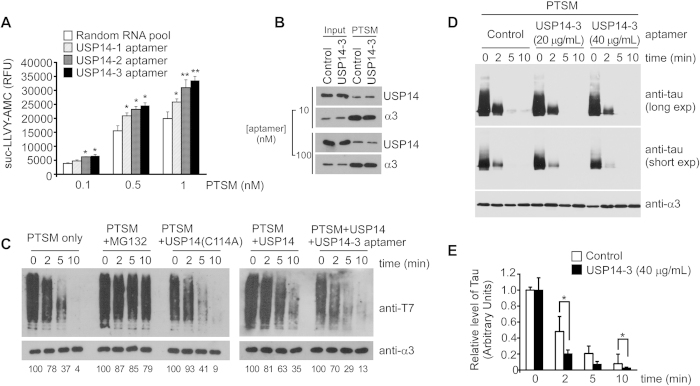
USP14 aptamers facilitate proteasomal degradation of polyubiquitinated substrates *in vitro*. (**A**) Proteasome activity was monitored by hydrolysis of the fluorogenic substrate suc-LLVY-AMC (12.5 μM) in the presence of aptamers (1 μg/mL). The random RNA pool was used as a control. *p < 0.05, **p < 0.01 (one-way analysis of variance with Bonferroni’s multiple comparison test). (**B**) The effect of USP14 aptamers on USP14 binding to the 26S proteasome was determined by immunoblotting after pulldown assays. (**C**) *In vitro* degradation assays using 20 nM polyubiquitinated T7-Sic1^PY^ (Ub-Sic1), PTSMs (5 nM), recombinant USP14 or USP14(C114A) (75 nM), and/or MG132 (1 μM) in the presence or absence of USP14-3 (20 μg/mL). Reaction mixtures at the indicated times were analyzed by SDS-PAGE and western blotting using antibodies against T7. Relative quantitation results are shown below. (**D**) *In vitro* Ub-tau degradation assay using PTSMs without USP14 reconstitution in the presence of USP14-3 (20 or 40 μg/mL). Ub-tau proteins were analyzed by SDS-PAGE and western blotting using antibodies against tau. Long exp, long exposure. Short exp, short exposure. Cropped gels/blots are used in the loading control panels of (**B**) and (**C**). (**E**) Quantitation of *in vitro* tau degradation in the presence or absence of USP14 aptamers. Data represent the mean ± SD from three independent experiments. *p < 0.05 (paired Student’s *t*-test).

Our results suggest that the RNA aptamers bind to USP14 and inhibit its deubiquitinating activity. We previously reported that inhibition of USP14’s Ub-chain trimming effects might enhance the proteolytic activities of proteasomes *in vitro* and *in vivo*^7^. To explore the possibility that inhibitory USP14 aptamers might enhance proteasome activity, we used suc-LLVY-AMC, a fluorogenic reporter substrate of 26S proteasomes. The fluorescent intensity as a result of suc-LLVY-AMC hydrolysis gradually increased over time (Fig. 3A and Supplementary Fig. 5). Proteasomal activity was higher when the USP14 RNA aptamers were present than when control aptamers were used, indicating that inhibiting USP14 with an RNA aptamer results in facilitated proteasomal degradation. Potentially USP14 aptamers inhibit USP14 by preventing its docking on the proteasome. However, direct pulldown assays indicated that the scenario is negative (Fig. 3B), indicating that the inhibitory effect by the RNA aptamer is mainly on USP14’s DUB activity.

We also used physiologically relevant polyubiquitinated proteins to simultaneously monitor proteolysis and Ub chain-trimming. Sic1, a cyclin-dependent kinase inhibitor in *Saccharomyces cerevisiae*, was polyubiquitinated using UBA1, UBC4, and RSP5 as cognate E1, E2, and E3 enzymes, respectively, along with other reconstitution components such as Ub and ATP^23^. The resulting Ub-Sic1 was gradually degraded when purified proteasomes were added. Addition of the proteasome inhibitor MG132 mutant protein in the reaction delayed Ub-Sic1 degradation (Fig. 3C). Mixing purified proteasomes with catalytically inactive recombinant USP14(C114A) facilitated the degradation of Ub-Sic1. In the control reaction, approximately 70% of Ub-Sic1 proteins were degraded by proteasomes after 10 min. However, in the presence of USP14 aptamers, Ub-Sic1 was almost completely degraded under the same conditions, showing similar kinetics for proteasomal degradation as USP14(C114A) was added in the reaction. These results suggest that USP14 aptamers function by inhibiting the catalytic activity of USP14, representing a unique biochemical and therapeutic potential for enhancing proteasome function.

Dysfunction in the UPS is closely related with tau degradation and neurodegeneration in AD^28^,^29^. Tau proteins are thought to be degraded by the UPS, especially during the early phases of tauopathy or AD progression^30^. In the human AD brain, proteasome activity is impaired^28^, and might be related to the age-dependent decrease of proteasome activity. However, it could also be the consequence of an accumulation of tau protein. To verify that proteasome activation by USP14 aptamers facilitates tau degradation, we prepared Ub-tau. The ability of proteasomes to degrade Ub-tau was enhanced in the presence of the USP14 aptamers (Fig. 3D,E). These results strongly suggest that inhibition of USP14 by RNA aptamers might antagonize Ub chain-trimming on proteasomes, consequently facilitating the degradation of many UPS substrates.

### USP14 aptamers enhanced tau degradation in cultured cells and protected against oxidative stress

**Figure 4:**
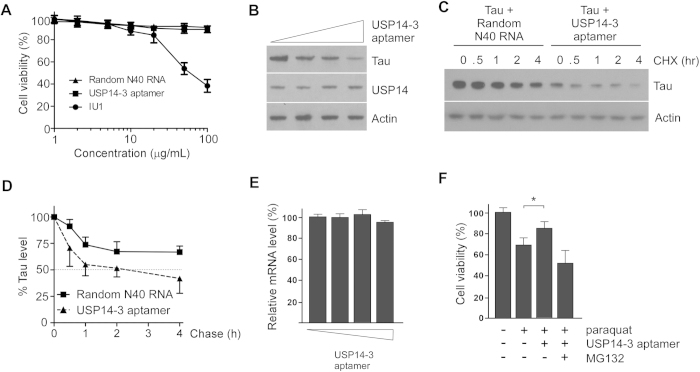
USP14 aptamers facilitated tau degradation and protected cells from tau-mediated cytotoxicity. (**A**) MTT assays were used to assess the cytotoxicity of USP14-3 aptamers on HeLa cells. (**B**) Inducible tau cell lines were treated with doxycycline (Dox, 250 pg/mL) for 24 h and then transfected with USP14-3 aptamers (0, 25, 50, or 100 nM). Levels of soluble tau proteins were analyzed by SDS-PAGE and western blotting. (**C**) Cycloheximide (CHX) chase analysis was used to examine the effects of USP14-3 on tau degradation. Inducible tau cell lines were transfected with the indicated aptamer (100 nM), treated with 250 pg/mL Dox for 24 h, and then 80 μg/mL CHX. Random 40 nt RNA oligonucleotides (100 nM) were used as controls. Cropped gels/blots are used. (**D**) Quantitation of tau levels in (C), which were normalized to actin. Data represent the mean ± SD from three independent experiments. (**E**) Post-translation tau regulation by USP14 aptamers. Quantitative reverse transcription PCR assays were performed using primers specific for tau and glyceraldehyde 3-phosphate dehydrogenase (GAPDH). Values indicate the means ± SD from three independent experiments. (**F**) Tau was induced with 250 pg/mL Dox. Oxidative stress was induced with 1 mM paraquat for 3 h prior to transfection with USP14-3 or random 40 nt RNA controls (100 nM each). Values are presented as the mean ± SD from five independent experiments. *p < 0.01 (one-way analysis of variance with Bonferroni’s multiple comparison test).

We investigated the effects of USP14 aptamers on HeLa cell viability. The small molecule USP14 inhibitor IU1 exhibited significant cytotoxicity at 50 μg/mL (~167 μM), while the USP14 aptamers exhibited no noticeable cytotoxicity at concentrations up to 100 μg/mL (Fig. 4A). The effect of the USP14 aptamers on the proteasomal degradation of tau was examined using a HEK293-derived cell line expressing the longest isoform of human tau (htau40) upon induction with doxycycline^25^. These cells expressed htau40 in a dose-dependent manner and produced SDS-resistant tau aggregates after about 2 days in culture^31^. When USP14-3 aptamers were transfected into cells, the levels of induced tau proteins were significantly decreased in a dose-dependent manner (Fig. 4B). The turnover of tau protein was facilitated by USP14-3 aptamers, as shown in our CHX chase analysis (Fig. 4C,D). Under those conditions, tau mRNA levels were comparable (Fig. 4E), indicating that accelerated tau degradation occurs post-translationally. The USP14 aptamers reduced cytotoxicity, which was induced by oxidative stress, in the presence of induced tau (Fig. 4F). Treatment with the proteasome inhibitor MG132 reversed the protective effects of USP14 aptamers. These results indicate that USP14 aptamer-induced proteasome activation might protect cells under various stressful conditions, including neurodegeneration. Future work is necessary to determine whether the RNA aptamers we identified can delay the formation of tau aggregates in the mouse brain.

## Discussion

We identified three RNA aptamers that specifically bind to USP14, a proteasome-associated DUB. These USP14-specific aptamers effectively inhibited deubiquitinating activity and enhanced proteasome activity *in vitro*. Cells treated with the aptamers showed facilitated degradation of soluble tau, delayed accumulation of tau aggregates, and enhanced cellular resistance to proteotoxic stress (Fig. 4). Treatment of cells with IU1 and USP14 aptamers yielded similar results, suggesting that activation of cellular proteasomes is possible through USP14 inhibition. RNA aptamers have some advantages over small molecules as therapeutic agents, and our findings suggest that USP14 aptamers could be used in the treatment of diseases associated with abnormal proteasome function.

Mammalian proteasomes contain three distinct DUBs: RPN11, USP14, and UCH37. RPN11 is a metalloprotease, and a constituent component of the RP. USP14 and UCH37 are cysteine proteases that are reversibly associated with RPN1 and RPN13 respectively, their cognates on the RP. It is thought that the 26S proteasome regulatory subunit RPN11 promotes substrate degradation, while other DUBs delay degradation. RPN11 cleaves the base of a polyUb chain and enables the substrate to enter the CP for proteolysis. USP14 and UCH37 trim Ub chains from the distal end of polyUb, thus decreasing the affinity of the chain for Ub receptors. USP14 has been reported to be capable of non-catalytically inhibiting proteasomes^32^, although the molecular mechanisms responsible for this are unclear.

In general, USP14 and UCH37 ensure that short or non-degradable Ub chains from substrates are released from the proteasome. The small molecule inhibitor of USP14, IU1, was identified by high-throughput screening, and found to enhance proteasomal degradation of target substrates *in vitro* and *in vivo*^7^*. In vitro* assays using Ub-Sic1 and Ub-tau with USP14 aptamers (Fig. 3C,D) indicated that elevated proteasomal activity might be due to catalytically inhibiting Ub chain-trimming of the proteasome substrate. The effects of USP14 aptamers on *in vitro* Ub degradation were more prominent when proteasomes were saturated with recombinant USP14 than when they were not. The different responses of Ub-Sic1 and Ub-tau to USP14 aptamers suggests that chain-trimming may not be a universal mechanism for regulating the rate of protein turnover. The weak response of tau proteins to USP14 inhibition suggests that their Ub chains have a higher binding affinity to the 26S proteasome compared with those of Sic1^23^. The USP14 aptamers enhance proteasomal degradation; however, it remains to be determined what features of Ub substrates, or whether the geometric morphology of polyUb chains affects degradation.

The *in vivo* stability of RNA aptamers can be improved by chemical modifications, while their biodistribution and clearance can be enhanced by conjugation with chemical moieties, such as polyethylene glycol or cholesterol. Aptamers were first reported in the early 1990s^9^ and have received widespread attention as potential therapeutic agents. The aptamer GB1-10 has been shown to recognize a glioblastoma-associated tenascin-C isoform, which is a useful marker for disease activity^33^. The expression of RNA aptamers against Ku protein in MCF-7 breast carcinoma cells potentially sensitized them to the anticancer drug etoposide^34^. The aptamer pegaptanib slows vision loss in people with neovascular age-related macular degeneration^35^. It has been approved by the United States Food and Drug Administration, and has been incorporated into European guidelines. Several other RNA and DNA aptamers have been developed for use in preclinical and clinical trials^19^. The USP14 aptamer could be used for modulating proteotoxic proteins in cells. The essential sequence and structure of USP14 aptamers that mediates the strong interactions with USP14 requires elucidation. This would assist in determining how these aptamers can be further truncated and modified without adversely affecting their binding activity *in vivo*.

Enhancing proteasome activity could be a therapeutic strategy to treat diseases caused by the accumulation of damaged and misfolded proteins, such as neurodegenerative diseases and cardiomyopathies. Li *et al.* showed that cellular proteasome levels were increased when the 11S proteasome was upregulated in cardiomyocytes. This enhanced proteasome-mediated removal of oxidized proteins, and protected cells against cardiac proteinopathy and myocardial ischemia^36^,^37^. In the current study, we observed that inhibiting USP14 with specific aptamers did not appear to alter proteasome levels, but alleviated tau- and paraquat-induced cytotoxicity. Our results indicate that enhanced proteasome activity can have a beneficial effect on cell viability under conditions of general proteotoxic stress. It remains to be determined whether USP14-specific RNA aptamers, or their modified forms, can accelerate the degradation of other proteopathic proteins. It was previously shown that IU1 was able to accelerate the degradation of tau, TDP43, ataxin 3, and glial fibrillary acidic protein^7^. The application of USP14 aptamers is not necessarily limited to cytoprotective roles against toxic protein accumulation. USP14 also plays an essential role in recycling Ub monomers, and in the dynamic regulation of Ub pools in cells^6^,^38^. Therefore, USP14 aptamers could be used to understand molecular mechanisms of USP14 activity and Ub homeostasis in cells.

## Additional Information

**How to cite this article**: Lee, J. H. *et al*. Facilitated Tau Degradation by USP14 Aptamers via Enhanced Proteasome Activity. *Sci. Rep.* **5**, 10757; doi: 10.1038/srep10757 (2015).

## Supplementary Material

Supplementary Information
