# Supplementary material for: Facilitated Tau Degradation by USP14 Aptamers via Enhanced Proteasome Activity
Source: Sci Rep. 2015 Jun 4;5:10757. doi: 10.1038/srep10757 (PMC4455164; doi:10.1038/srep10757)
Supplement: Supplementary Information [file srep10757-s1.pdf]

Supplementary Information

Facilitated Tau Degradation by USP14 Aptamers via Enhanced Proteasome Activity

Jung Hoon Lee, Seung Kyun Shin, Yanxialei Jiang, Won Hoon Choi, Chaesun Hong, Dong-Eun Kim, Min Jae Lee

SUPPLEMENTARY FIGURES AND LEGENDS

Supplementary Figures 1 to 5

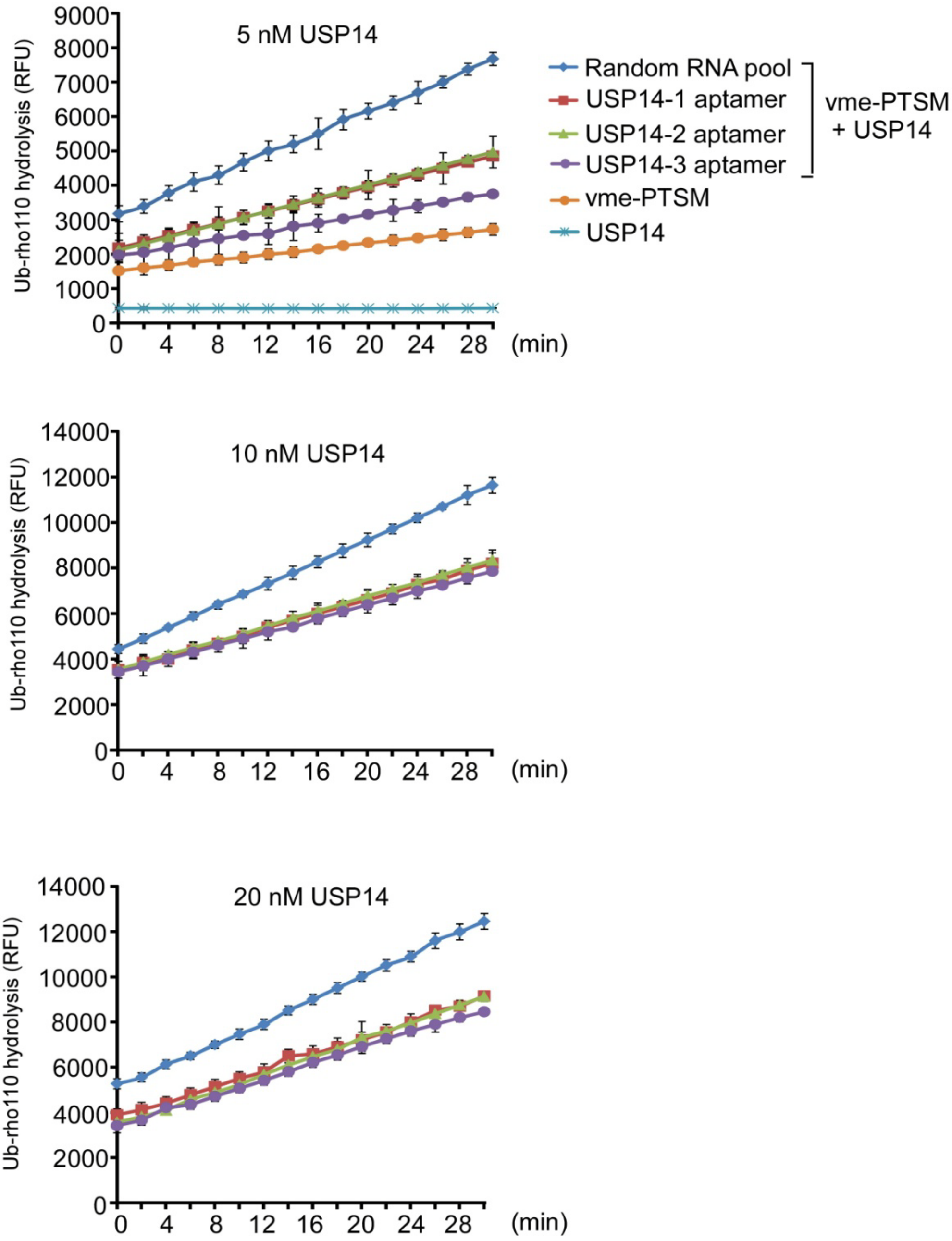

Supplementary Figure 1. The kinetic Ub-rho110 hydrolysis assay using various concentrations of recombinant USP14 proteins. Ub-rho110 (100 nM), ubiquitin-vinylmethylester-treated 26S human proteasomes (vme-PTSM, 1 nM) and 1 μg/mL of indicated aptamers (or random RNA pool controls) were used in a reaction. RFU, relative fluorescence units. These data complement Fig. 2D.

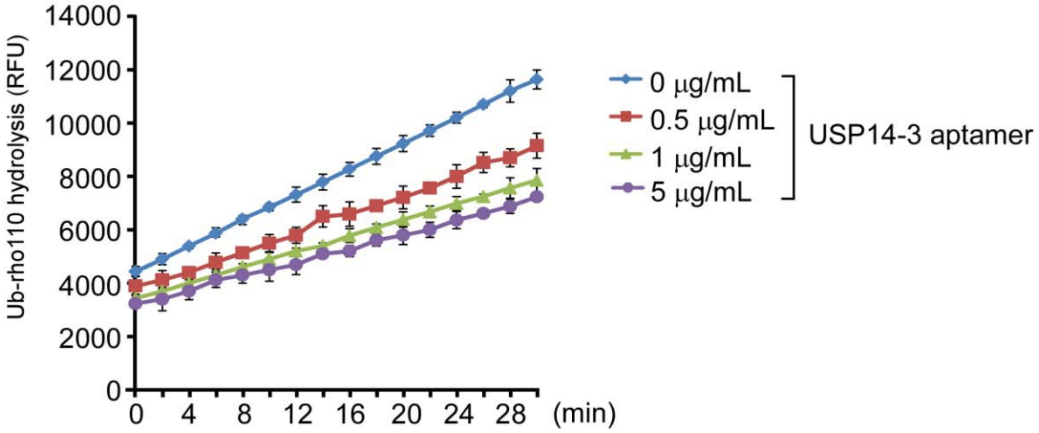

Supplementary Figure 2. The kinetic Ub-rho110 hydrolysis assay using various concentrations of USP14-3 aptamers (0, 0.5, 1, or 5 μg/mL), Ub-rho110 (100 nM), USP14 (10 nM), and vme-PTSM (1 nM). These data complement Fig. 2F.

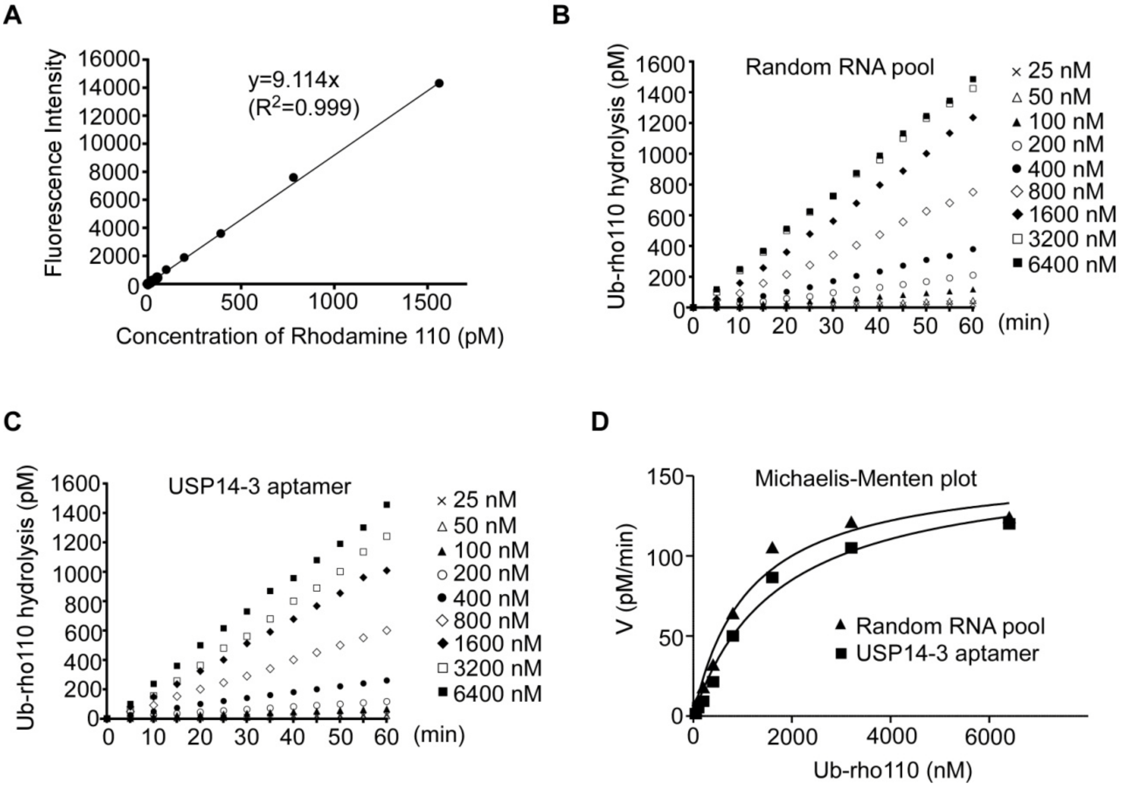

Supplementary Figure 3. Kinetic analysis of recombinant USP14 in the presence and absence of its apamers. (**A**) The standard curve of free rhodamine 110 to normalize fluorescence intensity ($R^2 > 0.99$). (**B**) Linear kinetics of Ub-Rhodamine 110 (Ub-rho110) hydrolysis by recombinant USP14 (10 nM) in the presence of vme-proteasomes (1 nM) and random RNA pools (1 μg/mL, equivalent to 33 nM). The graphs shown are representative of at least three independent experiments, and each data point is the mean ± SD of triplicate determinations. (**C**) As in (**B**) except that USP14-3 aptamers were used instead of random RNA pools. (**D**) Michaelis-Menten plot of concentration-dependent Ub-rho110 cleavage with or without aptamers for 60 min. The data are fit into Michaelis-Menten equation by nonlinear regression ($R^2 > 0.97$) (GraphPad Prism). Approximate K_M and k_{cat} were determined as 1124

± 248 nM and 0.938 ± 0.073 sec^{-1}, respectively, for control RNA and 1716 ± 300 nM and 0.948 ± 0.079 sec^{-1}, respectively, for USP14-3 aptamers. The graphs shown are representative of at least three independent experiments, and each data point is the mean of triplicate determinants.

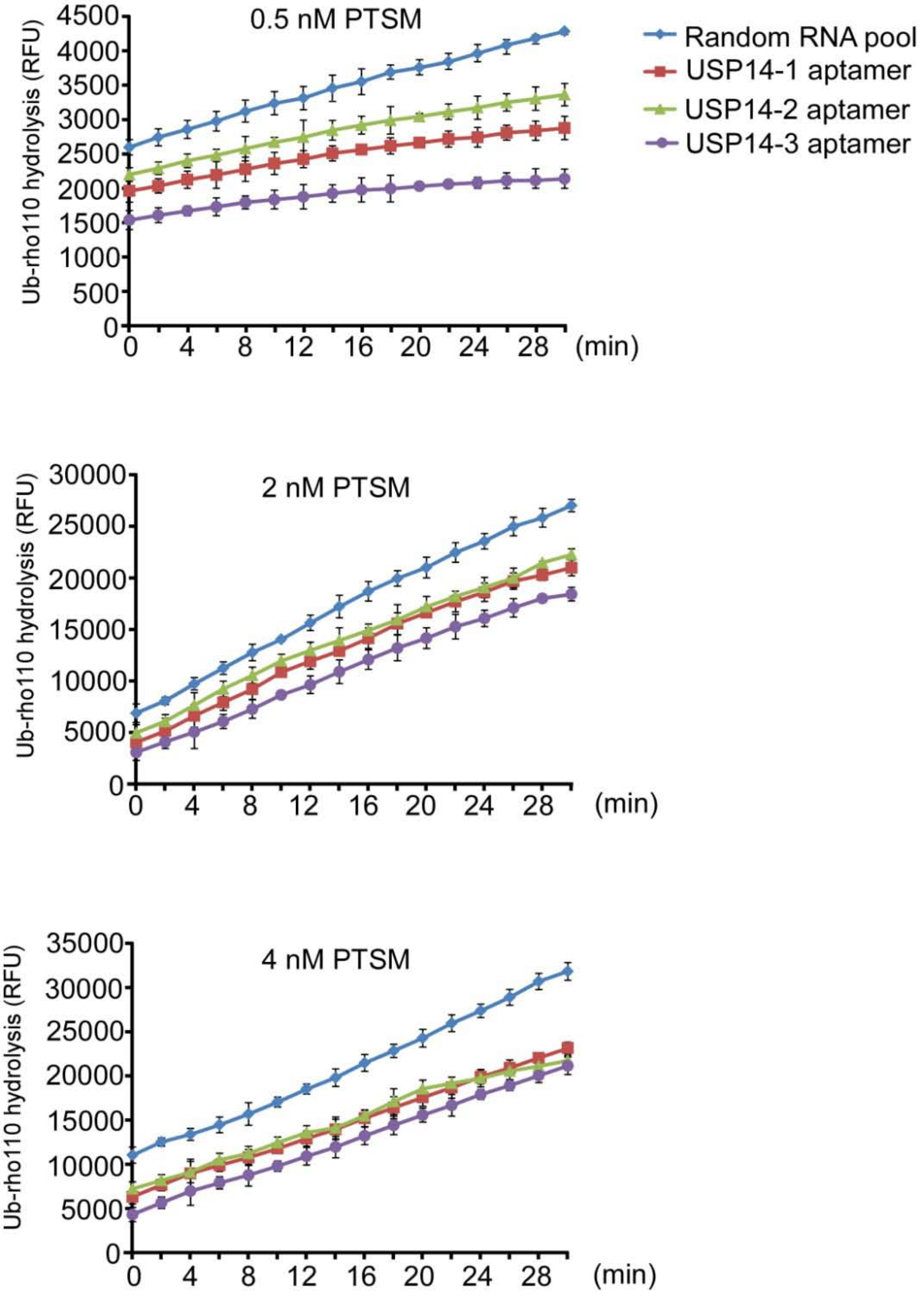

Supplementary Figure 4. The kinetic Ub-rho110 hydrolysis assay using various concentrations of purified human proteasomes (PTSM). Ub-rho110 (20 nM) and 1 μg/mL of indicated aptamers were used. These data complement Fig. 2G.

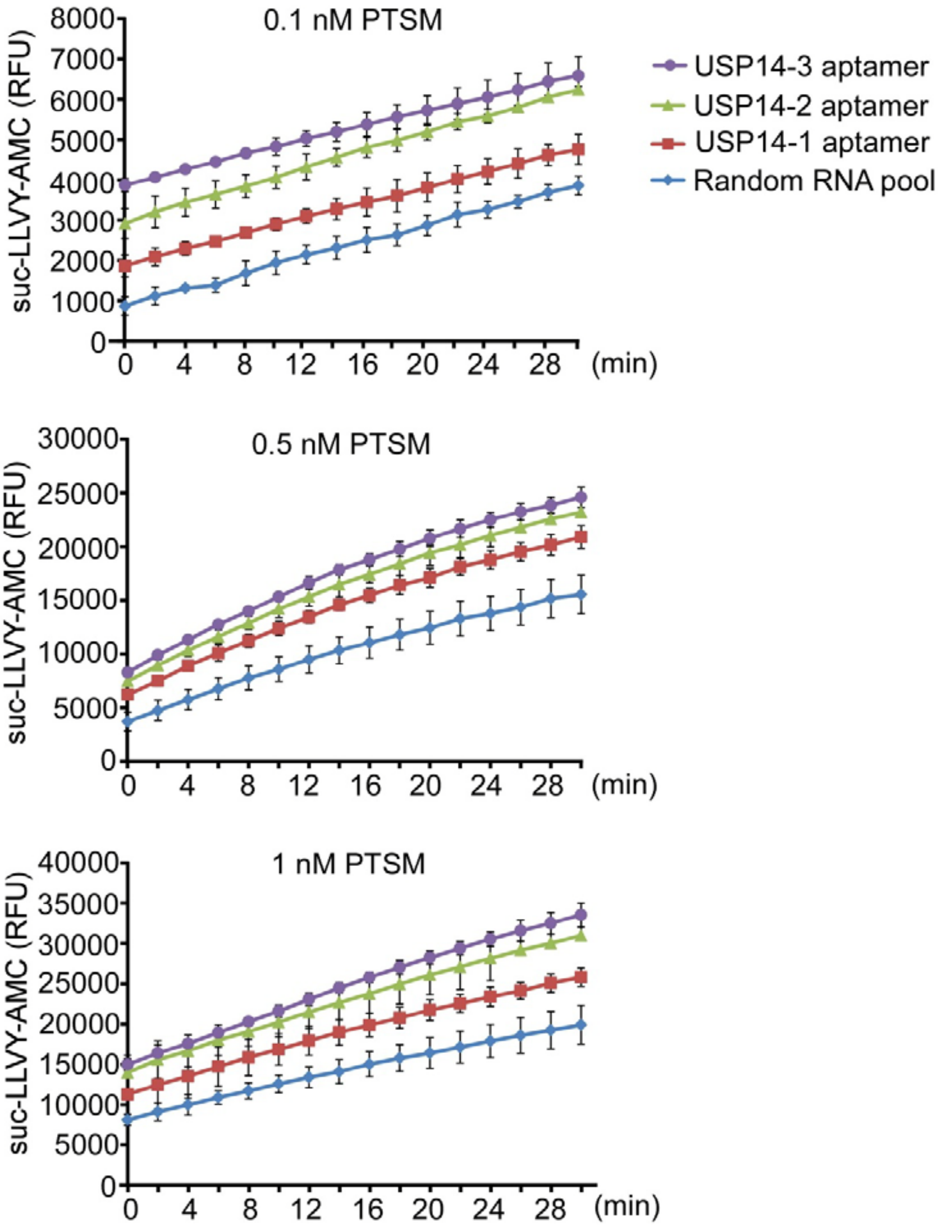

Supplementary Figure 5. The kinetic suc-LLVY-AMC hydrolysis assay using various concentrations of vme-untreated proteasomes (PTSM), suc-LLVY-AMC (12.5 μM), and 1 μg/mL of indicated aptamers in a reaction. These data complement Fig. 3A.
